# Reappraising the claims of evolutionary continuity between thanatosis and near-death experiences

**DOI:** 10.1093/braincomms/fcag368

**Published:** 2026-09-30

**Authors:** Jorge A Delgado-Ron

**Affiliations:** Faculty of Health Sciences, Simon Fraser University, Burnaby, British Columbia V5A 1S6, Canada

I read with interest the article by Peinkhofer *et al*.^1^ ‘The evolutionary origin of near-death experiences: a systematic investigation’, in *Brain Communications,* which sought to establish that near-death experiences (NDEs) and thanatosis are heritable behavioural traits serving the biological purpose of survival.^1^

NDEs are generally described as subjective experiences.^2,3^ Despite recent attempts to map neural mechanisms, no specific neural correlate has been found.^4^ In this scenario, a key epistemological limitation is that NDEs, by definition, can only be appraised through language. For now, we suspect some animals might undergo similar processes. However, we have yet to confirm that animal species have NDEs.

Thanatosis, on the other hand, was first defined in the animal kingdom and the question to whether humans preserved such a mechanism is open to interpretation. Tonic immobility (TI) is a defining characteristic of thanatosis, and humans do exhibit TI when attacked by another person, particularly in the context of sexual assault.^5,6^ Unlike NDEs, it seems reasonable to assume TI as a form of thanatosis that would allow for cross-comparison across species, including humans.

When considered *in tandem*, the epistemological limitations around these two concepts suggest that any meaningful comparison between NDEs and thanatosis can mostly occur through the examination of both phenomena in humans. Such a comparison can take many forms, including the systematic examination of common causes, characteristics, genetic signatures, or short- and long-term effects.

Following best practices in research, Peinkhofer *et al*.^1^ specified their research plan in advance through the Open Science Framework.^7^ The authors’ innovative approach combined a systematic review of the literature [work package (WP) 1] with retrospective and prospective data collection (WP 2 to 5). However, the registration falls short in describing the data analysis plan for this new data. For the systematic review, they clarify methods for identifying studies and establishing a cladogram for thanatosis. However, for the rest of WP—which is where NDEs and TI can be compared—the authors solely provided case definitions, which is consistent with building a case-series, but not with inferential goals. Finally, they described their study as ‘observational’, but its design only specifies descriptive statistics.

Given the above considerations, the methodology is appropriate to map thanatosis as an evolutionary trait, but it is ill-equipped to establish associations between TI and NDEs. It simply lacks an analysis plan. Moreover, the specific goal is described as ‘establish a line of argumentation’, which is consistent with hypothesis generation but not with inferential goals.

Despite the limited scope of the methodology, the authors discuss their findings as if it were inferential. For example, the authors sustain that ‘these two conditions [thanatosis and NDEs] not only share important features but are related’. Given the lack of formal methods to compare both conditions, the methodology of such a comparison is left as an exercise to the reader. The implicit messaging in the article is that a common cause would show an association. In other words, if a predator attack triggers an NDE, this suggests NDEs are a form of thanatosis. Based on this criterion, WP1 does not allow for NDE-TI comparison, WP2 shows that only 14% of NDEs are associated with ‘predator’ attacks (including inanimate objects). Finally, if we follow the predefined case definition and search criteria for WP3, WP4 and WP5, no evidence was found.

The authors supplemented this lack of results through an unplanned cursory Google search. Based on five narrative accounts, they conclude that ‘humans under attack by big animals … can both exhibit thanatosis and have NDEs’. However, the lack of case definition and quality assurance for these additional examples makes it unclear whether they should be counted as cases. Some of the narratives feature strong literary elements that suggest they might not be veridical and their inclusion as cases might require additional steps that verify the veracity and accuracy of these five anecdotes.

Another implicit line of argumentation is that NDEs and thanatosis share a common outcome: survival. WP1 does not address this argument directly as neither of the included articles of TI in humans^5,6^ conducted a survival analysis. Similarly, the authors did not conduct formal tests within their case-series [WP2 + Google search]. Based on the lack of formal analyses, affirming that ‘thanatosis is associated with a survival advantage in … humans’ also seems premature.

A broader issue with this systematic investigation is that the same data are used with two goals: hypothesizing and testing the hypothesis. If we are looking for examples where A is associated with B, building a database of such examples is not the best playground to test this association. A broader approach would be required where counterfactuals are present and considered *in tandem*.

Whereas the authors acknowledge that the vast majority of NDEs do not feature predators, they rationalize this lack of association by arguing NDEs are likely a residual trait. The alternative explanation—that NDEs and thanatosis are not related—is never truly addressed. Yet, a careful review of their own evidence suggests this is the most parsimonious explanation.

TeBockhorst *et al*.^5^ develop a thorough description of the TI phenomenology. In their subjects, TI seems to be partially induced as a form of defensive avoidance. The subjects reported, for example, actively ignoring the attacker’s gaze either by closing their eyes or through intense focus on other objects (e.g. clocks and mirrors). They also report pronounced feelings of fear at the outset of the attack, which weakens as dissociation occurs. During the event, the subjects were also fully aware of their surroundings, maintaining awareness. An avoidant personality is also associated with TI.

In contrast, NDEs are involuntary experiences^4^ whereas a minority of people report NDEs as distressing, the vast majority of them start with overwhelming feelings of peace and joy. While NDEs feature reports of hyperawareness, this is disconnected from the environment, with the exception of out-of-body reports. No reliable psychological profile has been developed for NDEs. Martial *et al*.^2^ hypothesize dissociation and fantasy proneness are risk factors, but even they acknowledge these correlations have exclusively been assessed after the NDE.^2^ Given NDEs are comparable to psychedelic experiences,^8^ psychological traits are unlikely to remain as they were before the event.^9^ Finally, other phenomenological characteristics of NDEs such as life reviews, spiritual encounters, or altered perception of time^3^ are not reported during TI.

TI and NDEs are also different in their long-term effects. Whereas TI often results in increased risk of post-traumatic stress disorder (PTSD),^5,6^ NDEs typically result in enhanced psychological functioning, with increased sense of meaning, reduced fear of death and increased prosociality.^3^ As Martial *et al*.^2^ have noted on a separate publication,^2^ NDEs could be understood as an involuntary psychedelic experience,^8^ and psychedelic therapy is currently being proposed as a *treatment* for PTSD.^10^ In other words, the long-term effects of NDEs and TI are not only different, but the opposite of each other.

At the end of their article, Peinkhofer *et al*.^1^ affirm that they ‘have built a line of evidence suggesting thanatosis is the evolutionary foundation of NDEs’. Yet, as with previous examples, this statement seems premature. The lack of formal testing prevents inferential goals and a significant part of the evidence presented in the article not only does not support this conclusion but contradicts it. I would argue not only that thanatosis and NDEs are not related (phenomenologically, through common cause, or through their effects), but also that the hypothesis that one constitutes the evolutionary foundation of the other remains highly speculative.

## Data Availability

Data sharing is not applicable to this article as no new data were created or analysed.

## References

[1] Peinkhofer C, Martial C, Cassol H, Laureys S, Kondziella D. The evolutionary origin of near-death experiences: A systematic investigation. Brain Commun. 2021;3(3):fcab132. 10.1093/braincomms/fcab132 PMC8260963 34240053

[2] Martial C, Fritz P, Gosseries O, et al A neuroscientific model of near-death experiences. Nat Rev Neurol. 2025;21(6):297–311. 10.1038/s41582-025-01072-z 40159547

[3] Delgado-Ron JA . Near-death experiences. Can Med Assoc J. 2026;198(20):E780–E780. 10.1503/cmaj.252003 PMC13218601 42185006

[4] Greyson B, Pehlivanova M. A neuroscientific model of near-death experiences reconsidered. Psychol Conscious Theory Res Pract. Published online October 2025. doi:10.1037/cns0000448

[5] TeBockhorst SF, O’Halloran MS, Nyline BN. Tonic immobility among survivors of sexual assault. Psychol Trauma Theory Res Pract Policy. 2015;7(2):171–178. 10.1037/a0037953 25793694

[6] Kalaf J, Vilete LMP, Volchan E, et al Peritraumatic tonic immobility in a large representative sample of the general population: Association with posttraumatic stress disorder and female gender. Compr Psychiatry. 2015;60:68–72. 10.1016/j.comppsych.2015.04.001 25891640

[7] Kondziella D . Systematic investigation into the phylogenetic origins of near-death experiences. *OSF Registries*. [Registration v1] doi:10.17605/OSF.IO/E8G7H

[8] Timmermann C, Roseman L, Williams L, et al DMT models the near-death experience. Front Psychol. 2018;9:1424. 10.3389/fpsyg.2018.01424 PMC6107838 30174629

[9] McCulloch DEW, Grzywacz MZ, Madsen MK, et al Psilocybin-induced mystical-type experiences are related to persisting positive effects: A quantitative and qualitative report. Front Pharmacol. 2022;13:841648. 10.3389/fphar.2022.841648 PMC8959755 35355714

[10] Miller CE, Zoladz PR. Evaluating the potential for psilocybin as a treatment for post-traumatic stress disorder. J Pharmacol Exp Ther. 2025;392(1):100026. 10.1124/jpet.124.002237 39893004

